# Data and Supplemental information for predicting the thermodynamic stability of perovskite oxides using machine learning models

**DOI:** 10.1016/j.dib.2018.05.007

**Published:** 2018-05-08

**Authors:** Wei Li, Ryan Jacobs, Dane Morgan

**Affiliations:** Department of Materials Science and Engineering, University of Wisconsin-Madison, Madison, WI 53706, USA

## Abstract

To better present the machine learning work and the data used, we prepared a supplemental spreadsheet to organize the full training dataset prepared from DFT calculations, the individual elemental properties, the generated element-based descriptors derived from the elements present in each perovskite composition, and lists of the specific features selected and used our machine learning models. We have also provided supplemental information which contains additional details related to our machine learning models which were not provided in the main text (Li et al., In press) [1]. In particular, the supplemental information provides results on training and testing five regression models (using the same data and descriptors as the regression of *E*_hull_ in main text) to predict the formation energies of perovskite oxides. We provided source code that trains the machine learning models on the provided training dataset and predicts the stability for the test data.

**Specifications Table**

**Table:** 

Subject area	*Physics, Chemistry, Machine Learning*
More specific subject area	*Thermodynamic stability of perovskite oxides dataset and source code for training of machine-learning predictive model*
Type of data	*Spreadsheet, Source Code, Word Document*
How data was acquired	*Density Functional Theory Calculations**[2]**, MAGPIE Elemental Property Database**[3]**, Online resources*[4], [5]*(See Manuscript**[1]**Sec. 2.1)*
Data format	*Analyzed*
Experimental factors	*N/A*
Experimental features	*N/A*
Data source location	*N/A*
Data accessibility	*Data is with this article*

**Value of the data**

• Provide thermodynamic stability data of more than 1900 perovskite oxides, which can be future extended to other data mining / machine learning tasks.
• Present an informative elemental property list for all elements in the periodic table, which can be valuable for building descriptors for other materials informatics projects.
• Provide detailed information on the feature selection and model training results, making it easy for other researchers to study feature importance, and to understand and reproduce the results in the manuscript.
• Provided source code for training machine learning models on provided training dataset and making predictions on new perovskite compounds. Users can supply their own perovskite dataset for training or testing.

## 1 Data

### 1.1 Project_complete_dataset.xlsx:

This spreadsheet contains 5 sheets, which are listed below.

1. DFT Calculated Dataset: The dataset of DFT-calculated E_hull_ and formation energies of 1929 perovskites.
2. Elemental Property Table: The 82 elemental properties of all elements in the periodic table.
3. Shannon Radius: The Shannon radii of the elements that appeared in the 1929 perovskites which were used to calculate geometric descriptors unique to the perovskite structure.
4. Dataset with Generated Features: The 962 descriptors (including 171 constant descriptors that were not used and 791 descriptors that were used for feature selection) generated from the elemental properties.
5. Top features Selected: The descriptors selected during feature selection as optimal (top 70 from the 791 descriptors) for use the regression model and the classification model.

### 1.2 Supplemental information.docx

This file contains additional details related to our machine learning models which were not provided in the main text. In particular, the supplemental information provides results on training and testing five regression models (using the same data and descriptors as the regression of *E*_hull_ in main text) to predict the formation energies of perovskite oxides. The supplemental information contains 4 sections: Models/APIs used in this work, Best Parameters for Each Model Investigated, Regression of Formation Energy, Learning Curve with the Best Model for Stability Classification.

### 1.3 Code repository

This repository contains source code for training/testing machine learning models on the provided dataset. This package can provide predictions of stability of new perovskite compounds using the machine learning approaches developed in this work. The code repository is available online at https://github.com/uw-cmg/perovskite-oxide-stability-prediction.

## 2 Experimental design, materials and methods

N/A.

## References

[1] W. Li, R. Jacobs, D. Morgan, Predicting the thermodynamic stability of perovskite oxides using machine learning models, 2018. Comp. Mater. Sci, **150** (2018) 454-463. 10.1016/j.dib.2018.05.007 PMC5992996 29892644

[2] Jacobs R. (2018). Material discovery and design principles for stable, high activity perovskite cathodes for solid oxide fuel cells. Adv. Energy Mater..

[3] Ward L. (2016). A general-purpose machine learning framework for predicting properties of inorganic materials. Npj Comput. Mater..

[4] N. Herr The Sourcebook for Teaching Science. 2007 (cited accessed May 2017); Available from: 〈https://www.csun.edu/science/ref/spreadsheets/〉.

[5] Shannon Rt (1976). Revised effective ionic radii and systematic studies of interatomic distances in halides and chalcogenides. Acta Crystallogr. Sect. A: Cryst. Phys. Diffr. Theor. Gen. Crystallogr..

