# Supplementary material for: Data and Supplemental information for predicting the thermodynamic stability of perovskite oxides using machine learning models
Source: Data Brief. 2018 May 8;19:261–3. doi: 10.1016/j.dib.2018.05.007 (PMC5992996; doi:10.1016/j.dib.2018.05.007)
Supplement: Supplementary file 1 — Supplementary material [file mmc3.docx]

**Electronic Supplemental Information: Predicting the thermodynamic stability of perovskite oxides using machine learning models**

Wei Li^1^, Ryan Jacobs^1^, Dane Morgan^1^

^1^ Department of Materials Science and Engineering, University of Wisconsin-Madison, Madison, Wisconsin, 53706, USA.

# S1 Models/APIs used in this work

**Table S1** Summary of models/APIs used in this work from scikit-learn.

| **Models/API name** | **Description** |
| --- | --- |
| sklearn.linear_model.LinearRegression | Ordinary least squares Linear Regression |
| sklearn.kernel_ridge.KernelRidge | Kernel ridge regression |
| sklearn.tree.DecisionTreeRegressor | A decision tree regressor |
| sklearn.neural_network.MLPRegressor | Multi-layer Perceptron regressor |
| sklearn.ensemble.ExtraTreesRegressor | An extra-trees regressor |
| sklearn.feature_selection.RFE | Feature ranking with recursive feature elimination |
| sklearn.linear_model.RandomizedLogisticRegression | Randomized Logistic Regression |
| sklearn.model_selection.ShuffleSplit | Random permutation cross-validator |
| sklearn.linear_model.LogisticRegression | Logistic Regression (aka logit, MaxEnt) classifier |
| sklearn.svm.SVC | Support Vector Machines Classifier |
| sklearn.tree.DecisionTreeClassifier | A decision tree classifier |
| sklearn.neural_network.MLPClassifier | Multi-layer Perceptron classifier |
| sklearn.ensemble.ExtraTreesClassifier | An extra-trees classifier |
| sklearn.feature_selection.mutual_info_classif | Estimate mutual information for a discrete target variable |
| sklearn.model_selection.StratifiedShuffleSplit | Stratified ShuffleSplit cross-validator |
| sklearn.preprocessing.scale | Standardize a dataset along any axis |

# S2 Best Parameters for Each Model Investigated

See perovskite_DFT_EaH_FormE.xlsx for complete dataset, continuous_descriptors.csv and discrete_descriptor.csv for continuous feature matrix and discrete feature matrix, respectively.

**Table S2** Summary of model performance with hyperparameters optimized only on included data written as (score +/- error), where the score and error are calculated as the mean test score on excluded data, and 2×standard deviation of 5 random leave-out 20% stratified splits, respectively.

| **Classifier Model** | **Logistic Regression** | **SVM with RBF kernel** | **Decision Tree** | **Neural Network** | **Extra Trees** |
| --- | --- | --- | --- | --- | --- |
| Accuracy | 0.817 (+/- 0.026) | 0.927 (+/- 0.028) | 0.882 (+/- 0.020) | 0.927 (+/- 0.022) | 0.932 (+/- 0.019) |
| Precision | 0.648 (+/- 0.039) | 0.889 (+/- 0.074) | 0.805 (+/- 0.026) | 0.886 (+/- 0.063) | 0.900 (+/- 0.058) |
| Recall | 0.825 (+/- 0.072) | 0.857 (+/- 0.051) | 0.791 (+/- 0.076) | 0.862 (+/- 0.053) | 0.865 (+/- 0.055) |
| F_1_ score | 0.726 (+/- 0.043) | 0.872 (+/- 0.047) | 0.792 (+/- 0.040) | 0.874 (+/- 0.036) | 0.882 (+/- 0.033) |
| **Regression Model** | **Linear Regression** | **Kernel Ridge with RBF** | **Decision Tree** | **Neural Network** | **Extra Trees** |
| R^2^ | 0.748 (+/- 0.072) | 0.912 (+/- 0.024) | 0.751(+/- 0.108) | 0.896 (+/- 0.037) | 0.909 (+/- 0.017) |
| RMSE (meV/atom) | 43.5 (+/- 3.8) | 25.7 (+/- 2.2) | 43.0 (+/- 7.6) | 27.9 (+/- 2.7) | 26.1 (+/- 1.8) |
| MAE (meV/atom) | 31.7 (+/- 2.8) | 16.5 (+/- 1.4) | 24.9 (+/- 2.8) | 18.4 (+/- 1.2) | 15.2 (+/- 0.8) |

**Table S3** Summary of optimized hyperparameters used in the classification and regression models.

| **Classifier Model** | **Logistic Regression** | **SVM with RBF kernel** | **Decision Tree** | **Neural Network** | **Extra Trees** |
| --- | --- | --- | --- | --- | --- |
| **Parameters** | penalty='l2', C=0.1706, class_weight='balanced', solver='liblinear' | kernel='rbf', gamma = 0.025, C=31.6 | criterion='entropy', max_depth=22,  max_features=20, min_impurity_split= 0.00336,  class_weight='balanced' | activation='logistic', solver='lbfgs', alpha=0.7, hidden_layer_sizes=(60) | criterion='entropy', bootstrap= False,  min_impurity_split=0.1, max_features=43, class_weight='balanced',  min_samples_split=5, max_depth= 18, n_estimators=115 |
| **Regression Model** | **Linear Regression** | **Kernel Ridge with RBF** | **Decision Tree** | **Neural Network** | **Extra Trees** |
| Parameters for E_hull_ | N/A | kernel='rbf', gamma=0.007, alpha=0.007 | criterion='mse', max_depth=12, max_features=30,  min_impurity_split=0.0785 | activation='logistic', solver='sgd', alpha=0.545, hidden_layer_sizes=(68), max_iter=700 | min_samples_split = 5, criterion = 'mse', n_estimators= 189, bootstrap = False, min_impurity_split=0.1, max_features = 30, max_depth=20 |

Features ordered by importance from the recursive feature elimination selection method are shown in the files RFE_clf_order.txt, RFE_eah_order.txt for classification of stability, and regression of energy above convex hull values, respectively.

**Table S4** Description of the top-selected elemental properties.

| **Elemental Property** | **Property Definition** |
| --- | --- |
| Number of unfilled valence orbitals | Number of unfilled valence orbitals |
| Coefficient of thermal expansion | The coefficient of thermal expansion of the element |
| HHIp | Herfindahl−Hirschman Index Production Value |
| Mendeleev Number | Position on the periodic table, counting column-wise from hydrogen |
| BCC energy | the energy of the element in the Open Quantum Materials Database(OQMD) ground state crystal structures minus that of the BCC DFT value, all at 0 K |
| Group number | The IUPAC periodic group number of the element |
| BCC Fermi | The DFT-calculated BCC crystal structure Fermi level of the element |
| Number of d valance orbitals | Number of filled d valence orbitals |
| Heat of Vaporization | Heat of vaporization of the element |
| HHIr | Herfindahl−Hirschman Index Reserve Value |
| Third ionization potential | Third ionization potential (energy) of the element |

# S3 Regression of Formation Energy

Besides the classification of stability and regression of E_hull_ values shown in the paper, we also trained and tested five regression models (same as regression of E_hull_ in main text) to predict the formation energies of perovskite oxides. These formation energies represent the energy to form the oxide from the constituent elements, where the constituent elements are the DFT-calculated stable states at T=0 K in the Materials Project database. Here, we used the same set of perovskites as used in the E_hull_ analysis in the main text and calculated the DFT formation energies to generate the dataset. **Fig. S1** shows how the formation energies are distributed.


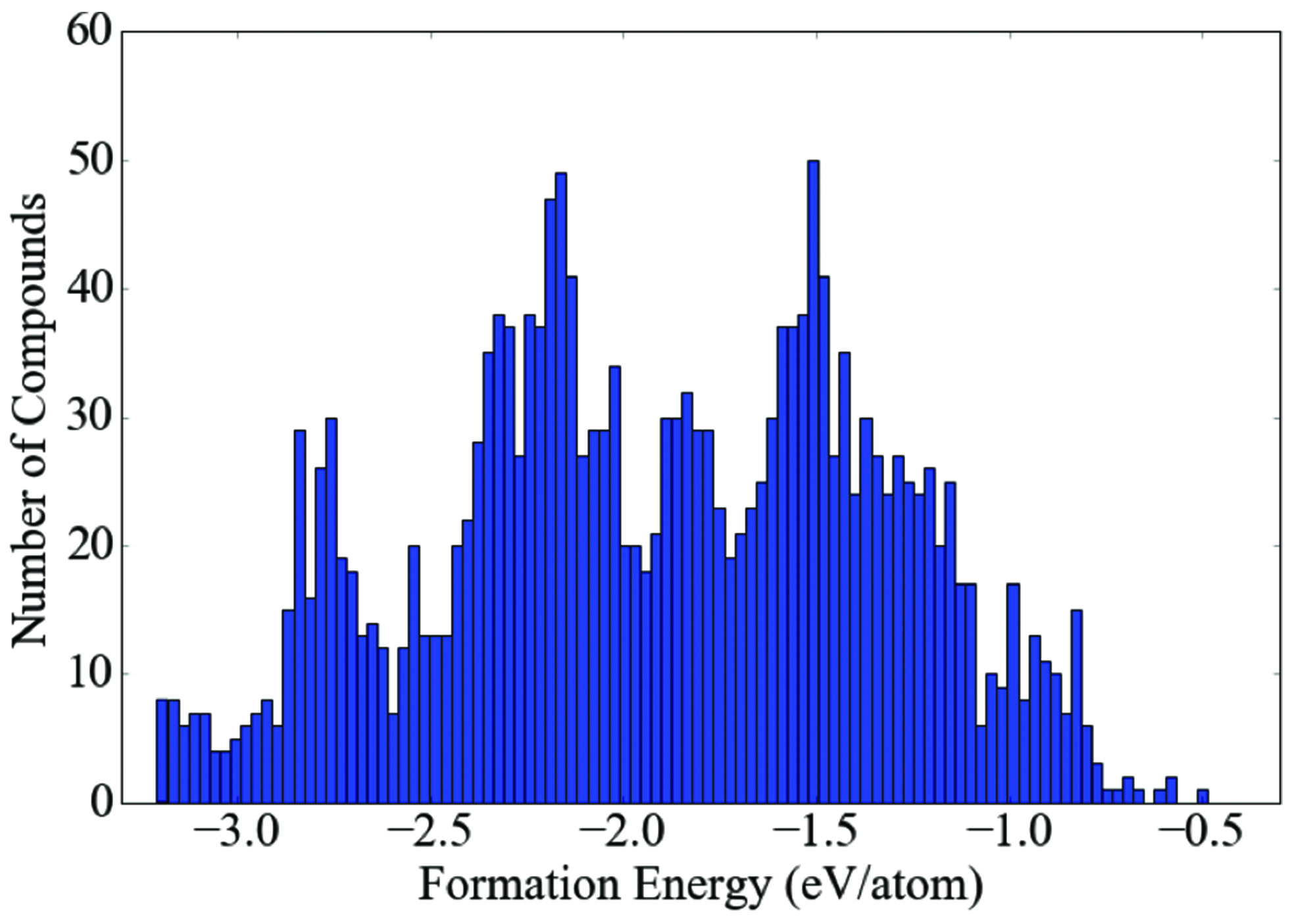


**Fig. S1** Histogram of formation energy of all 1929 perovskite compounds considered in this work.

We performed the feature selection and model selection in the same way described in the paper. **Fig. S2** shows the cross-validation score against increasing number of features selected by three feature selection methods. We selected stability selection as the best feature selection method and chose to use the top 20 features for model training based on the fact that the cross-validation R^2^ score decreased dramatically with number of features less than 20 but changed little for more than 20 features. The list of features selected is provided in the file stability_fe_order.txt. We tested five regression model on the selected top 20 features and tuned the parameters of each model based on the cross-validation R^2^ score. **Table S5** shows a summary of all optimized parameters used in each model. **Table S6** presents a comparison of R^2^, RMSE and MAE values between five regression models for prediction of formation energy. The best model we found is kernel ridge with rbf kernel with RMSE of 0.062 eV/atom and MAE of 0.032 eV/atom. **Fig. S3** plots the fitted formation energy values against the DFT calculated values, which shows a good approximation of formation energy via machine learning approach.


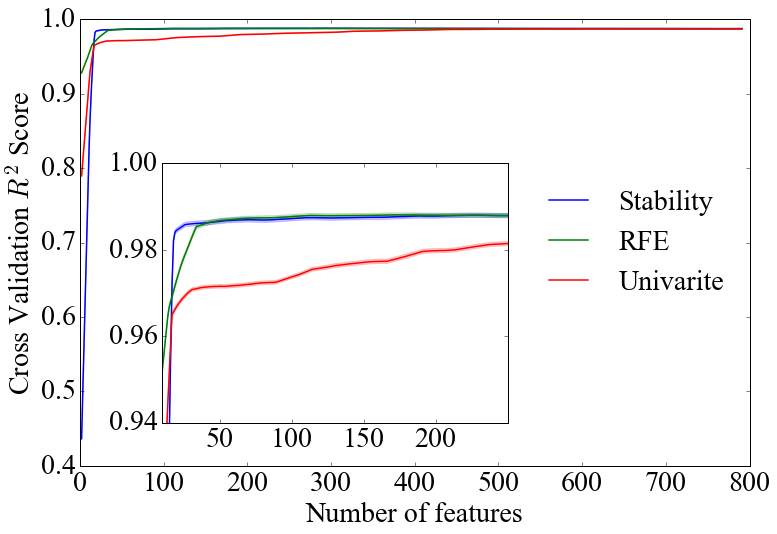


**Fig. S2** Value of the cross validation R^2^ score with increasing number of features three selection methods in regression task: stability selection, recursive feature elimination (RFE) and univariate selection based on mutual information. The R^2^ score is calculated as the average of 20 random splits of leave out 20% cross-validation.

**Table S5** Summary of optimized parameters used in each model.

| **Regression Model** | **Linear Regression** | **Kernel Ridge with RBF** | **Decision Tree** | **Neural Network** | **Extra Trees** |
| --- | --- | --- | --- | --- | --- |
| Parameters for formation energy | N/A | kernel='rbf',gamma=0.0215, alpha=0.00464 | criterion='mse', max_depth=42, max_features=None, min_impurity_split=0.0016 | activation='logistic', solver='lbfgs', alpha=1, hidden_layer_sizes=(66), max_iter=700 | criterion='mse', bootstrap=False, max_leaf_nodes=None,  min_impurity_split=0.005, max_features=8,  min_samples_split=2, min_samples_leaf=1, max_depth=17, n_estimators=135 |

**Table S6** Comparison of R^2^, RMSE and MAE values between five regression models for prediction of formation energy. The values are listed as (score +/- error), where score and error are calculated as the mean value, and 2×standard deviation of 20 random splits of leave out 20% cross-validation, respectively.

| **Model** | | **Linear Regression** | **Kernel Ridge with RBF** | **Decision Tree** | **Neural Network** | **Extra Trees** |
| --- | --- | --- | --- | --- | --- | --- |
| **Prediction Evaluation Metrics** | R^2^ | 0.965 (+/- 0.008) | 0.988 (+/- 0.007) | 0.959 (+/- 0.012) | 0.973 (+/- 0.008) | 0.983 (+/- 0.006) |
|  | RMSE (eV/atom) | 0.107 (+/- 0.012) | 0.062 (+/- 0.019) | 0.116 (+/- 0.021) | 0.094 (+/- 0.012) | 0.074 (+/- 0.014) |
|  | MAE (eV/atom) | 0.072 (+/- 0.007) | 0.032 (+/- 0.004) | 0.069 (+/- 0.010) | 0.063 (+/- 0.006) | 0.047 (+/- 0.006) |


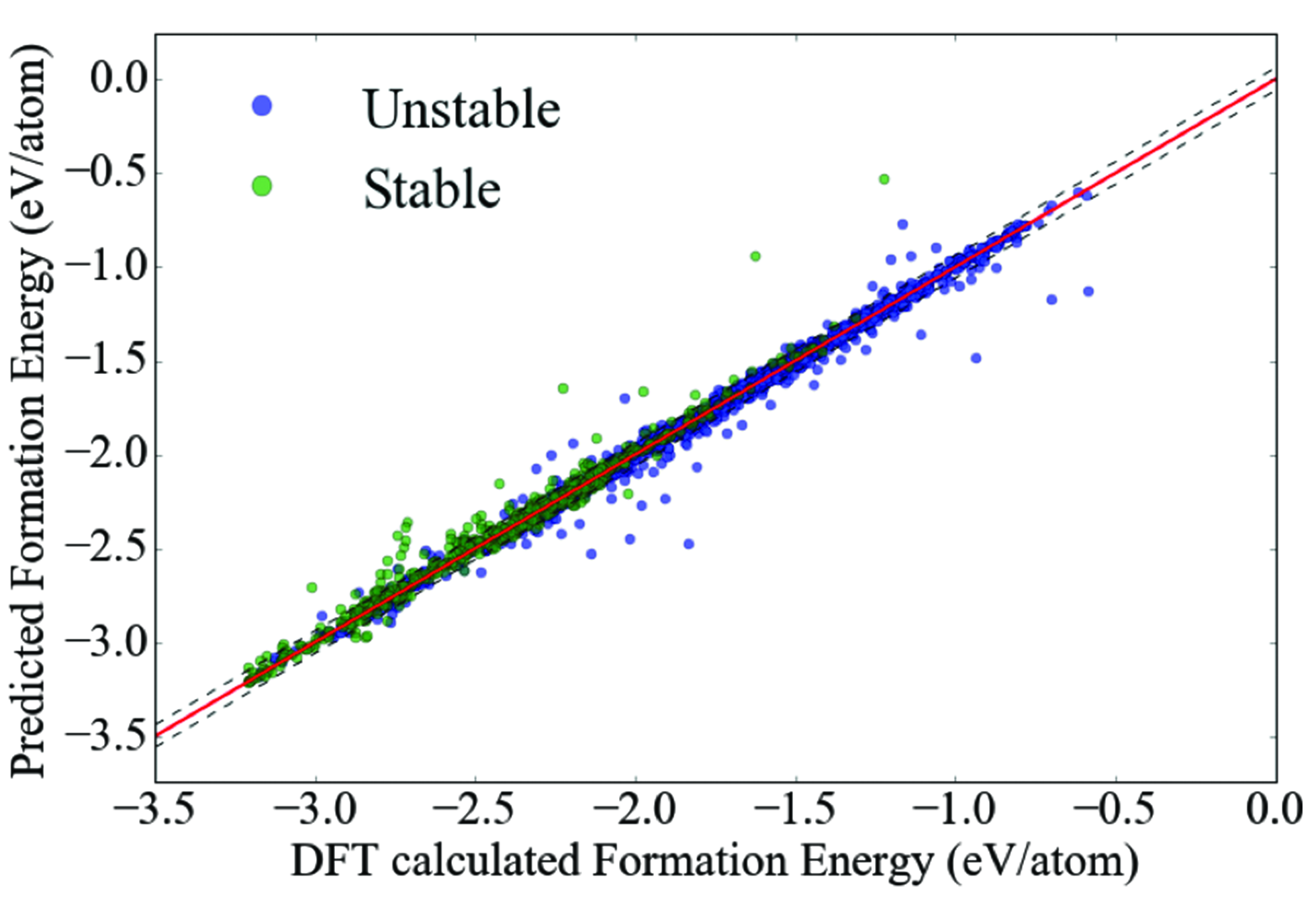


**Fig. S3** Fitted formation energy values versus the DFT calculated values. The green and blue points represent materials that were found to be stable and unstable based on DFT calculations, respectively. The y=x line is shown as a solid red line and the surrounding dashed lines represent +/- error (in **Table S4**) shifts from the y=x line.

# S4 Learning Curve with the Best Model for Stability Classification

In addition to the ROC curve, we evaluated our extra trees classifier by plotting its learning curve, as shown in **Fig. S4**. **Fig. S4** shows the cross-validation F_1_ score of the extra trees model plotted against the number of compounds (training examples) used in the dataset (For each F1 value on this plot, we randomly selected the number of compounds specified by the x coordinate as a leave-out test set from total dataset and found its F1 score, then repeated this 20 times, and then averaged the 20 F_1_ scores). When the number of training samples is too small, the model over-fits the training dataset. The cross-validation F_1_ score increases as the number of training examples grows, which indicates more training examples could provide more information of the physics governing the thermodynamic stability of perovskites.


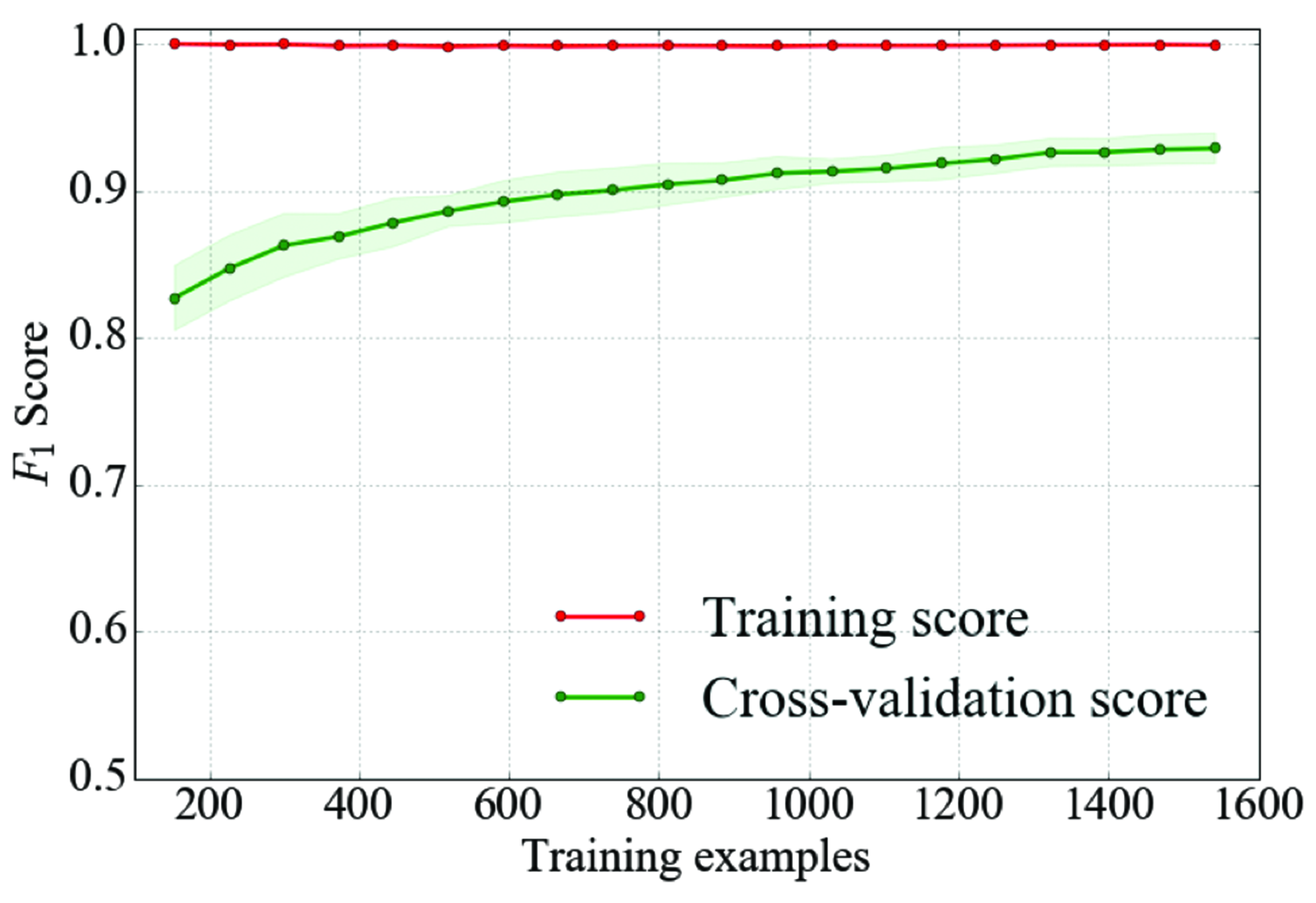


**Fig. S4** The learning curve of extra trees classifier with F_1_ score plotted against the number of training examples used. Training score and cross-validation score are calculated as the average score of 20 random splits on the training set and the testing set, respectively.
