# Supplementary material for: Data and Supplemental information for predicting the thermodynamic stability of perovskite oxides using machine learning models
Source: Data Brief. 2018 May 8;19:261–3. doi: 10.1016/j.dib.2018.05.007 (PMC5992996; doi:10.1016/j.dib.2018.05.007)
Supplement: Supplementary file 2 — Supplementary material [file mmc1.docx]

**Conflicts of interest**

There are no conflicts to declare.
